# The Role of Mitochondrial DNA in Mediating Alveolar Epithelial Cell Apoptosis and Pulmonary Fibrosis

**DOI:** 10.3390/ijms160921486

**Published:** 2015-09-07

**Authors:** Seok-Jo Kim, Paul Cheresh, Renea P. Jablonski, David B. Williams, David W. Kamp

**Affiliations:** 1 Department of Medicine, Division of Pulmonary and Critical Care Medicine, Jesse Brown VA Medical Center, Chicago, IL 60612, USA; E-Mails: seokjo.kim@northwestern.edu (S.-J.K.); p-cheresh@northwestern.edu (P.C.); renea.jablonski@northwestern.edu (R.P.J.); D-Williams@northwestern.edu (D.B.W.); 2 Division of Pulmonary & Critical Care Medicine, Northwestern University Feinberg School of Medicine, Chicago, IL 60611, USA

**Keywords:** mitochondrial DNA damage, oxidative stress, Sirtuin 3, alveolar epithelial cell, pulmonary fibrosis

## Abstract

Convincing evidence has emerged demonstrating that impairment of mitochondrial function is critically important in regulating alveolar epithelial cell (AEC) programmed cell death (apoptosis) that may contribute to aging-related lung diseases, such as idiopathic pulmonary fibrosis (IPF) and asbestosis (pulmonary fibrosis following asbestos exposure). The mammalian mitochondrial DNA (mtDNA) encodes for 13 proteins, including several essential for oxidative phosphorylation. We review the evidence implicating that oxidative stress-induced mtDNA damage promotes AEC apoptosis and pulmonary fibrosis. We focus on the emerging role for AEC mtDNA damage repair by 8-oxoguanine DNA glycosylase (OGG1) and mitochondrial aconitase (ACO-2) in maintaining mtDNA integrity which is important in preventing AEC apoptosis and asbestos-induced pulmonary fibrosis in a murine model. We then review recent studies linking the sirtuin (SIRT) family members, especially SIRT3, to mitochondrial integrity and mtDNA damage repair and aging. We present a conceptual model of how SIRTs modulate reactive oxygen species (ROS)-driven mitochondrial metabolism that may be important for their tumor suppressor function. The emerging insights into the pathobiology underlying AEC mtDNA damage and apoptosis is suggesting novel therapeutic targets that may prove useful for the management of age-related diseases, including pulmonary fibrosis and lung cancer.

## 1. Introduction

Pulmonary fibrosis is characterized by an over abundant accumulation of extracellular matrix (ECM) collagen deposition in the distal lung interstitial tissue in association with an injured overlying epithelium and activated myofibroblasts. Idiopathic pulmonary fibrosis (IPF) is the most common variety of lung fibrosis and carries a sobering mortality approaching 50% at 3–4 years [1]. Although many of the cellular and molecular mechanisms underlying the pathophysiology of lung fibrosis have emerged from numerous studies over the past several decades, the precise pathways involved, their regulation, and the role of crosstalk between cells are not fully understood. With the exception of two FDA-approved drug therapies (pirfenidone and nintenanib) emerging in the fall of 2014, there are no effective therapies for patients with IPF. Furthermore, these two drugs primarily slow disease progression rather than improve lung function or symptoms. A better understanding of the pathobiology of pulmonary fibrosis is critically important in the design of more useful therapies.

**Figure 1:**
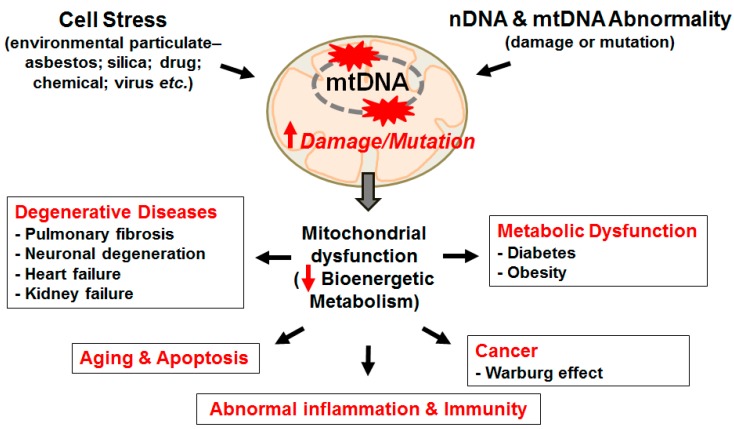
Hypothetical model whereby mtDNA damage induces diverse degenerative diseases. MtDNA damage and mutation can be induced by cell stress from environmental particulates and/or DNA abnormality. MtDNA damage/mutation cause mitochondrial dysfunction reducing bioenergeneric metabolism that can promote degenerative diseases, metabolic dysfunction, aging, apoptosis and cancer. Red-up arrow, increase; red-down arrow, decrease.

**Figure 2:**
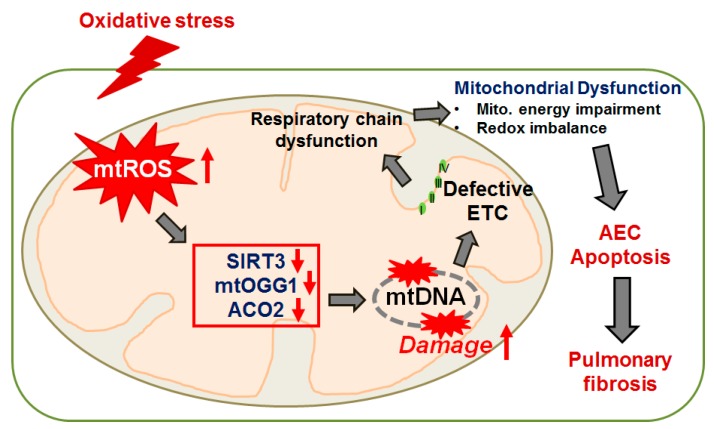
Proposed model of mtDNA damage in mediating AEC intrinsic apoptosis and pulmonary fibrosis. Oxidative stress-induced mtROS induces mtDNA damage by decreasing SIRT3, ACo-2 and mtOGG1 in AEC. MtDNA damage causes a defective ETC that can promote mitochondrial dysfunction, AEC apoptosis, and pulmonary fibrosis. I, II, III, IV, four different complex of ETC in mitochondria. Red-up arrow, increase; red-down arrow, decrease.

As will be reviewed herein, the extent of alveolar epithelial cell (AEC) injury, repair, and aging are emerging as critical determinants underlying pulmonary fibrosis. The purpose of this review is to highlight our current understanding of the causal role of AEC mitochondrial DNA (mtDNA) damage following oxidative stress in promoting AEC apoptosis and pulmonary fibrosis. Although oxidative mtDNA damage in other cell types (*i.e.*, vascular endothelial cells, macrophages, fibroblasts, *etc.*) are likely important, we concentrate on the lung epithelium given its prominent role in the pathophysiology of lung fibrosis. In particular, we focus on asbestosis (pulmonary fibrosis arising following asbestos exposure) as it shares radiographic and pathologic features with IPF though IPF is more common and carries a worse prognosis. Our group is using the asbestos paradigm to better understand the pathophysiologic mechanisms underlying pulmonary fibrosis. We begin with a brief overview of the evidence implicating that oxidative stress induces mtDNA damage and thereby promotes AEC apoptosis and pulmonary fibrosis. We explore the evidence that mitochondrial-derived reactive oxygen species (ROS) trigger an AEC mtDNA damage response and apoptosis that can promote lung fibrosis and other degenerative lung diseases (*i.e.*, lung cancer and chronic obstructive pulmonary disease [COPD]). We discuss the emerging role for AEC mtDNA damage repair by 8-oxoguanine DNA glycosylase (OGG1) and mitochondrial aconitase (ACO-2) in maintaining mtDNA integrity, which is important in preventing AEC apoptosis and asbestos-induced pulmonary fibrosis. We review the emerging evidence on the important crosstalk between mitochondrial ROS production, mtDNA damage, p53 activation, OGG1, and ACO-2 acting as a mitochondrial redox-sensor involved in mtDNA maintenance in animal models of lung fibrosis. We summarize recent studies linking the sirtuin (SIRT) family members, especially SIRT3, to mitochondrial integrity and mtDNA damage repair and aging. SIRT3 is considered the ‘guardian of the mitochondria’ because it is the major mitochondrial deactylase controlling mitochondrial metabolism playing important roles in mtDNA integrity and the prevention of aging. Finally, we present a conceptual model of how SIRTs modulate ROS-driven mitochondrial metabolism that may be important for cell survival as well as their tumor suppressor function. A general hypothetical model linking mtDNA damage and mitochondrial dysfunction to diverse degenerative diseases, including pulmonary fibrosis, aging, and tumorigenesis is shown in Figure 1. Specifically, herein we focus on a proposed model of AEC mtDNA damage in mediating AEC-intrinsic apoptosis and pulmonary fibrosis as illustrated in Figure 2. Collectively, these studies are revealing novel insights into the pathobiology underlying AEC mtDNA damage and apoptosis that should provide the rationale for developing novel therapeutic targets for managing age-related diseases such as pulmonary fibrosis, COPD, and lung cancer.

## 2. The Mitochondria, mtDNA, and ROS—The Basics

Mitochondria are maternally inherited and have an essential cellular function of generating energy in the form of ATP via respiration, hence are the “powerhouse” of the cell. Mitochondria also are critically important in regulating complex survival signals that determine whether cells live or die and are closely involved in additional functions, such as cellular differentiation, growth, and cell cycle control [2]. The number of mitochondria in a cell ranges from one to several thousand, a range largely determined by the tissue type and organism [3,4]. Human mtDNA is composed of 16,569 nucleotide bases and encodes 13 polypeptides of the electron transport chain (ETC), 22 transfer RNAs, and two ribosomal RNAs located in the inner mitochondrial membrane (IMM) matrix [4,5]. MtDNA, which encodes approximately 3% of all mitochondrial proteins, is present in multiple copies (~100) per cell, whereas nearly 1200 nuclear DNA (nDNA)-encoded mitochondrial proteins are translated in the cytosol and imported into the mitochondria [3,4,5]. Interestingly, the extent of mtDNA alterations that occurs during development affects the presence and emergence of mtDNA genetic variations and mutations primarily in a region termed the D-loop [6,7]. This D-loop on the mtDNA forms the basis of forensic medicine in human identification and has been a useful tool in molecular anthropological studies on human origins [7]. Some proteins encoded by nDNA have been shown to be essential for maintaining mtDNA integrity including OGG1, ACO-2, mitochondrial transcription factor A (Tfam), and others [8,9,10,11,12,13]. However, the mitochondrial proteins involved in mtDNA repair (mostly base excision repair [BER]) are all nuclear encoded and highly dependent on the nDNA repair machinery systems [14,15]. Notably, compared to nDNA, mtDNA is ~50-fold more sensitive to oxidative damage, in part due to its proximity to the ETC and ROS production, lack of a histone protective shield overlying the mtDNA, and relatively limited DNA repair mechanisms [12,13,16,17]. As compared to nDNA, oxidative stress-induced mtDNA damage has a mutation rate that is 10-fold greater [18,19,20,21,22,23]. Not surprisingly, mtDNA damage and subsequent mutations can lead to mitochondrial dysfunction, including the collapse in the mitochondrial membrane potential (ΔΨ_m_) and release of pro-apoptogenic agents which drives disease formation, aging, and tumorigenesis (Figure 1) [12,19].

ROS, such as hydroxyl radicals (•HO), superoxide anions (O_2_^•−^), hydrogen peroxide (H_2_O_2_), and others are primarily generated under physiologic conditions from the mitochondrial ETC but also from other intracellular sources [12,18,24,25,26,27,28,29]. Although low levels of ROS are important for promoting cell survival signaling pathways and antioxidant defenses, higher ROS levels, as occurs in the setting of disease or aging, cause oxidative damage to biomolecules, such as DNA, protein, and lipids. Oxidative DNA damage can result in apoptosis and senescence, tumorigenesis, and degenerative diseases ([7,12,18,24,25,26,28], Figure 1). Furthermore, mtDNA variants within cells can affect both energy and non-energy pathways (complement, inflammatory, and apoptotic) supporting a paradigm shift in thinking about the role of mitochondria beyond simply energy production [7,12,30].

Biological tissues, especially the lungs, are exposed to both extrinsic sources of ROS (e.g., tobacco, asbestos, silica, radiation, bleomycin, and other drugs) and intrinsic sources (such as those from inflammatory, mesenchymal, epithelial, and endothelial cells) primarily via the mitochondrial ETC as well as numerous enzyme systems including Nicotinamide adenine dinucleotide phosphate (NADPH) oxidases (NOXs), xanthine oxidase, and nitric oxide synthase (NOS) [12,19,31]. Intracellular ROS can also be generated from redox-active ferrous (Fe^2+^) iron within or on the surface of asbestos fibers via the Haber-Weiss reaction [31,32], resulting in the accumulation of •HO and other free radicals. As reviewed in detail elsewhere, several lines of evidence demonstrate that ROS play a role in pulmonary fibrotic disease: (1) oxidized lipids and proteins have been identified from the exhaled air, BAL fluid, and lung tissue of patients with fibrotic lung disease [33,34]; and (2) Bleomycin-induced pulmonary fibrosis (the most common animal fibrosis model) is associated with increased levels of ROS, oxidized proteins, DNA, and lipids [35,36]; (3) Increased oxidative DNA damage is seen in IPF, silicosis, and asbestosis patients, as well in experimental animal models of silicosis or asbestos-induced lung fibrosis [37]; (4) Antioxidants and iron chelators can attenuate fibrosis induced by bleomycin or asbestos in rodent models [35,38]. Additionally, there is some evidence implying that mitochondria-generated ROS of lung parenchymal cells mediate pulmonary fibrosis [36]. Exogenous toxins, such as asbestos fibers, can also induce mitochondrial ROS in lung epithelial cells and macrophages; both of which are important target cells implicated in pulmonary fibrosis (see for reviews: [31,35,37]). However, more work is needed to determine the precise molecular mechanisms involved as well as any cross-talk between cell types. A key role of alveolar macrophage (AM) mitochondrial ROS in mediating asbestosis has been suggested in the studies by Carter and colleagues [39,40,41,42]. These investigators showed that mitochondrial Rac-1 levels are elevated in AM from patients with asbestosis, that Rac-1 augments asbestos-induced AM H_2_O_2_ production, and that ROS production is reduced by knockdown of the iron-sulfur complex III in the mitochondrial ETC. These data implicate H_2_O_2_ production via electron transfer from Rac-1 to complex III may activate cellular injury pathways that promote asbestosis.

Although the precise role of H_2_O_2_-induced AEC mtDNA damage in mediating pulmonary fibrosis is unclear, a possible causal role for H_2_O_2_ in promoting lung fibrosis is supported by several lines of evidence that we recently reviewed in detail elsewhere [35], and briefly summarize herein; some of the key points are: (1) catalase, a H_2_O_2_ scavenger, blocks H_2_O_2_-induced human IPF fibroblast activation [34]; and prevents asbestos-induced fibrosis in rats [43,44]; (2) glutathione (GSH), an antioxidant, is diminished in IPF lungs and epithelial lining fluid [34,45,46]; and (3) although n-acetyl cysteine (NAC), a GSH precursor, attenuates bleomycin-induced fibrosis in rodents and increases lung GSH levels, NAC administration to patients with IPF was recently proven no better than placebo [47,48]. Similar to H_2_O_2_, considerable evidence reviewed elsewhere, implicates NOXs, especially NOX1, 2, and 4 isoforms, in the pathogenesis of pulmonary fibrosis, including AEC apoptosis and apoptosis-resistant myofibroblasts [49,50,51,52,53].

## 3. AEC Apoptosis and Lung Fibrosis—Role of the Mitochondria

### 3.1. AEC Aging, Apoptosis and Pulmonary Fibrosis

Accumulating evidence firmly implicate that “exaggerated” aging lung has a crucial role in the pathogenesis of lung fibrosis, although the detailed molecular mechanisms involved are not fully established (see for reviews: [1,54]). The nine proposed pivotal hallmarks mediating the “aging phenotype” include: genomic instability, telomere shortening, epigenetic alterations, deficient proteostasis, dysregulated nutrient sensing, mitochondrial dysfunction, cellular senescence/apoptosis, stem cell depletion, and distorted intercellular communication [55]. However, many of the nine aging pathways are implicated in humans with IPF, including AEC DNA damage, activation of epigenetic signaling, shortened alveolar type II (AT2—the distal lung epithelial stem cell) cell telomeres, AEC mitochondria-mediated (intrinsic) apoptosis, and activated endoplasmic reticulum (ER) stress response in apoptotic AECs (see for reviews: [1,37,54,56,57,58,59,60]). Herein we focus on mtDNA damage since it is an early event in oxidant-exposed cells that may contribute to the inflammatory, fibrogenic and malignant potential of asbestos [37,57,61,62]. Notably, genome-wide association studies (GWAS) have established an important role for aberrant DNA repair pathways in patients with IPF [55,63,64,65]. As reviewed in detail elsewhere [7,66], mutations in maternally-inherited mtDNA encoding for key genes of mitochondrial energy-generating oxidative phosphorylation, rather than Mendelian nuclear genetic principles, better accounts for the complex clinical-pathological features of many common degenerative and metabolic disease whose tissue stem cells are bioenergetically abnormal (Figure 1).

In contrast to catastrophic lytic/necrotic cell death that can trigger an inflammatory response, apoptosis is a regulated, ATP-dependent process of cell death that results in the elimination of cells with extensive DNA damage without eliciting an inflammatory response. Apoptotic cellular responses occur by two mechanisms: (1) the extrinsic (death receptor) pathway and (2) the intrinsic (mitochondria-regulated) pathway. Others [19,67] as well as ourselves [35,37] have extensively reviewed these pathways recently we, therefore, confine our comments to some of the more recent updates centered on mitochondrial dysfunction and mtDNA damage in driving AEC apoptosis.

Considerable evidence reviewed elsewhere convincingly demonstrates that AEC apoptosis is one of the key pathophysiologic events hindering normal lung repair and thus promoting pulmonary fibrosis [1,35,37,52,56,57,58,59,68,69,70,71]. Briefly summarizing some of the key findings includes the following: (1) patients with IPF and animal models of asbestos- and silica-induced pulmonary fibrosis show significant lung epithelial cell injury, ER stress and apoptosis; (2) fibrogenic dusts, such as asbestos and silica, can induce both lytic and apoptotic AEC death in part by generating ROS derived from the mitochondria or NOXs; (3) DNA damage, which is a strong activator of intrinsic apoptosis, occurs in the AEC of human patients with IPF and murine models of asbestos-induced lung fibrosis and can activate p53, an important DNA damage response molecule; (4) protein S-glutathionylation, in part through effects occurring in the ER, mediates redox-based alterations in the FAS death receptor important for triggering extrinsic lung epithelial cell apoptosis and pulmonary fibrosis; and (5) blocking αvβ6 integrin release from injured lung epithelial cells, prevents latent TGF-β activation and pulmonary fibrosis following radiation or bleomycin exposure.

Perhaps the most convincing evidence implicating AEC apoptosis in the pathophysiology of pulmonary fibrosis is that genetic approaches targeting apoptosis of Alveolar epithelial type 2 cell (AT2 cells) in mice and humans demonstrate an important role for AECs (see for reviews: [56,58]). A primary requirement for AEC death and inadequate epithelial cell repair in causing pulmonary fibrosis as first pointed out by Haschek and Witschi 35 years ago has now been elegantly verified by various transgenic murine models of pulmonary fibrosis and genetic mutations in 10 different surfactant protein C (SPC) BRICHOS domain mutations that are only evident in AT2 cells in humans with interstitial pulmonary fibrosis [56]. A clear genetic predisposition to developing IPF is evident in 5%–20% of patients [1,56,58,59,63,64,65]. Notably, many mutations associated with the development of pulmonary fibrosis are only expressed in epithelial cells (*i.e.*, surfactant C and A2 genes, MUC5b), while others are more ubiquitously expressed. Although a detailed discussion of these mutations is beyond the scope of this article, SPC mutations can induce AEC ER stress response that promotes AEC apoptosis and pulmonary fibrosis [59]. Among the most common gene mutations seen in patients with IPF and familial pulmonary fibrosis involve telomerase (TERT and TERTC). Unlike surfactant mutations, telomerase mutations are ubiquitously expressed in cells, especially in stem cells [72]. Shortened telomeres, which are associated with aging-related diseases due to oxidative stress, are evident in the majority of AT2 cells (the distal lung epithelial stem cells) in patients with lung fibrosis [72,73]. One study showed that AT2 cells from 97% of 62 IPF patients (both sporadic and familial) had shortened telomeres [73]. Although mitochondrial dysfunction and ROS production appear important in driving telomerase-dependent cell senescence, the role of mtDNA damage is uncertain [74]. The finding that AEC telomerase shortening, alone, does not trigger lung fibrosis, nor augment bleomycin-induced lung fibrosis in mice, strongly implicates other genetic and/or environmental factors are likely crucial [75].

### 3.2. Mitochondria-Regulated AEC Apoptosis

The intrinsic apoptotic death pathway is activated by various fibrotic stimuli (*i.e.*, ROS, DNA damage, asbestos, *etc.*) that stimulate pro-apoptotic Bcl-2 family members action at the mitochondria that results in increased permeability of the outer mitochondrial membrane, reduced ΔΨ_m_, and the release of numerous apoptotic proteins (*i.e.*, cytochrome c, *etc.*) that subsequently activate pro-apoptotic caspase-9 and caspase-3 (see for reviews: [19,35,37,58,67,68]). Notably, a crucial role for mtDNA damage in driving intrinsic apoptosis was established in cell-sorting studies demonstrating that persistent mtDNA damage results in the collapse in the ΔΨ_m_ and intrinsic apoptosis [76]. Bleomycin-induced fibrosis in mice is blocked in the pro-apoptotic Bid deficient mice [77]. Bid is activated by the death receptor pathway and triggers intrinsic apoptosis by blocking anti-apoptotic Bcl-2 molecules and thereby enabling Bax/Bak-mediated apoptosis [77]. However, additional studies are warranted to better understand how Bcl-2 family members modulate mtDNA integrity to impact AEC survival and prevent lung fibrosis. More recently, two groups have established that PTEN-induced putative kinase 1 (PINK1) deficiency impairs AEC mitochondrial function in patients with IPF and PINK1-deficient mice have increased AEC intrinsic apoptosis and lung fibrosis following viral-induced ER stress or bleomycin exposure [78,79]. Interestingly, the pro-fibrotic cytokine TGF-β may be protective to lung epithelial by promoting PINK1 expression and attenuating AEC apoptosis that drives lung fibrosis [79]. Collectively, these studies firmly support an important role of AEC mitochondria-regulated apoptosis in the pathophysiology of pulmonary fibrosis. Additional studies are required to further characterize the precise molecular mechanisms involved, the role of mtDNA damage, and crosstalk between AEC and macrophages. Furthermore, the translational significance of any identified targets in animals exposed to fibrogenic agents will need to be investigated in humans with IPF.

Others as well as our group have been using the asbestos paradigm to better inform our understanding of how oxidative stress resulting from fiber exposure promotes lung epithelial cell intrinsic apoptosis important in the development of pulmonary fibrosis. As we have extensively reviewed these studies from our group elsewhere [35,37], we summarize herein only some of the salient supporting findings including: (1) asbestos fibers are internalized by AECs soon after exposure, resulting in the production of iron-derived ROS, mtDNA damage, and intrinsic apoptosis as evidenced by decreased ΔΨ_m_, mitochondrial cytochrome c release into the cytosol, and activation of caspase-9 and 3 (but not caspase-8); (2) these deleterious actions by asbestos on AECs are blocked by phytic acid (an iron chelator), benzoic acid (a free-radical scavenger), and overexpression of Bcl-XL; and (3) an important role for mitochondrial ROS is suggested by the findings that asbestos-induced AEC intrinsic apoptosis and p53 activation are blocked in cells unable to produce mitochondrial ROS and that asbestos preferentially induces mitochondrial ROS production as assessed using a highly sensitive rho-GFP probes targeted to the mitochondria or cytosol. Furthermore, as reviewed below (see Section 3.3), we find a direct relationship between asbestos-induced mtDNA damage and intrinsic AEC apoptosis. Mossman and colleagues showed that activated protein kinase delta (PKCδ) migrates to the mitochondria of lung epithelial cells *in vitro* and *in vivo* following asbestos exposure and is crucial for promoting asbestos-induced mitochondria-regulated apoptosis and fibrosis via mechanisms dependent upon pro-apoptotic Bim activation [80,81]. Taken together, mitochondrial ROS production and PKCδ activation following asbestos exposure appear important for inducing p53 activation and intrinsic lung epithelial cell apoptosis. However, the role of mtDNA integrity in modulating p53 and PKCδ activation requires additional study.

### 3.3. mtDNA Damage and Repair—Role in Cancer and Lung Fibrosis

Prompt repair of damaged mtDNA is important given the accumulating evidence convincingly showing that mtDNA damage and mutations are linked to various pathologic conditions, including lung fibrosis (see for reviews: [7,12,18,19,82]). MtDNA mutations accumulate in tissues with aging in part via disruptions in mitochondrial quality control pathways. MtDNA deletions that occur early in development can become widely disseminated throughout the body and cause spontaneous mitochondrial dysfunction [7]. Further, mtDNA deletions [83] and mutations [84] can arise in cells of various tissues throughout life and their accumulation modulates aging and longevity [7,84]. These findings suggest that the accumulation of mtDNA mutations arising from mtDNA damage caused by aging, environmental exposure, and other forms of oxidative stress support the “mitochondrial theory of aging” that may be crucial in depleting the longevity of important stem cells (*i.e.*, AT2 cells in the distal lung) and promoting the pathobiology of degenerative diseases and tumorigenesis [3,7,12].

Cancer is characterized by altered energy metabolism (Warburg effect) arising from mtDNA mutations and changes in mtDNA copy number [85,86]. Of 41 human lung, bladder, and head and neck tumors examined, mutated mtDNA occurred 19–220 times more frequently than nDNA [87]. The importance of mtDNA mutations in lung cancer is supported by the observation that over 40% of patients with lung cancer demonstrate mutations in their mtDNA [88]. MtDNA mutations can compromise ETC function and contribute to altered metabolism driving accelerated aerobic glycolysis in the setting of metastatic progression [89]. Additionally, severe mtDNA damage promotes mitochondrial genome deletion [90]. Tumor cells lacking mtDNA can acquire mtDNA of host origin, resulting in sequential recovery of respiration from primary to metastatic tumor cells [91]. There is also evidence that mtDNA mutations can preferentially accumulate in non-small cell lung cancer (NSCLC) tissues as compared to matched blood samples [88]. Taken together, these studies demonstrate that mtDNA damage and mutations occur in malignant cells, including lung cancers, and that preservation of mtDNA integrity may be an innovative preventative therapeutic target.

Base excision repair (BER), which is the major mtDNA repair mechanism, has been reviewed in detail elsewhere [18]. All mtDNA repair proteins are nuclear-encoded and imported into mitochondria. 8OHdG, the most common of ~50 DNA base changes that occur with oxidative stress, is highly mutagenic in replicating cells by causing G:C→A:T transversions that can contribute to tumorigenesis and aging [12,18]. Mutations in the hOGG1 gene occur in patients with lung cancer and other malignancies [92]. OGG1 is over three-fold more active in the mitochondria as compared to the nucleus and *Ogg1^−/−^* mice have a 20-fold increase in liver mitochondrial 8OHdG levels [18,92]. Mitochondria-targeted OGG1 (mt-OGG1) over-expression prevents mitochondria-regulated apoptosis caused by oxidative stress, including AEC apoptosis following asbestos exposure [8,9,12,13,62,93,94]. OGG1 has two isoforms (α and β), yet curiously the βOGG1 isoform has negligible DNA repair activity despite being in 50-fold excess as compared to the αOGG1 isoform in the mitochondria [95]. This suggests that βOGG1 isoform plays a role in mitigating mitochondrial oxidative stress independent of its BER activity. We recently showed that overexpression of mt-OGG1 or mt-OGG1 mutants incapable of DNA repair promote AEC survival despite high levels of asbestos-induced mitochondrial ROS stress [9]. Although one mechanism by which mt-OGG1 preserves mitochondrial function is by increasing mtDNA repair, we identified a novel function of OGG1 in chaperoning mitochondrial aconitase (ACO-2) from oxidative degradation and, thereby, preserving mtDNA integrity [9]. ACO-2, a mitochondrial tricarboxylic acid cycle (TCA) enzyme, is a sensitive marker of oxidative stress and, notably, preserves mtDNA in yeast independent of ACO-2 activity [96,97]. We also reported that oxidative stress (asbestos or H_2_O_2_) preferentially induces mtDNA than nuclear DNA damage in AEC both *in vitro* as well as *in vivo* and that OGG1 preservation of ACO-2 is crucial for preventing asbestos-induced AEC mtDNA damage, intrinsic apoptosis, and pulmonary fibrosis [8,98]. Additional support for a protective role of ACO-2 is that ACO-2 inactivation has been linked to decreased lifespan in yeast and progressive neurodegenerative diseases in humans [99]. Thus, ACO-2 appears to have a dual function in the TCA cycle for mitochondrial bioenergy production as well as for preserving mtDNA. The precise molecular mechanisms by which mt-OGG1 and ACO-2 interact to preserve AEC mtDNA integrity are not fully understood. Furthermore, additional studies are necessary to assess the translational significance of AEC OGG1 and ACO-2 in preserving mtDNA integrity and preventing pulmonary fibrosis. 

### 3.4. Animals Models of Pulmonary Fibrosis—Role of Mitochondrial ROS, mtDNA Damage, and Mitochondrial Dysfunction

Accumulating evidence reviewed above strongly implicates mitochondrial ROS production, mtDNA damage, and mitochondrial dysfunction (in part due to PINK1 deficiency) in the pathophysiology of AEC apoptosis and pulmonary fibrosis. Herein we review some of the other more recent animal lung fibrosis models that have better informed our understanding of the field. Gadzhar *et al.* [36] examined adult Wistar rat lungs at various time points after a single intratracheal dose of bleomycin and observed lung fibrosis, as measured by Ashcroft scores, collagen, and TGF-β levels at day 14. Evidence of ROS, as assessed by malondialdehyde (MDA) production, was noted as early as 24 h after bleomycin treatment and continued to increase over 14 days. By day seven, mtDNA deletions were significantly elevated and disruption of the mitochondrial architecture as assessed by electron microscopy was noted in lung tissue. Notably, these mitochondrial abnormalities resulted in dysfunction of ETC subunits encoded by mtDNA, but not nDNA, and mtDNA deletions and mtDNA-encoded ETC dysfunction were directly associated with pulmonary TGF-β levels that were predictive of developing lung fibrosis in a multivariate model.

As pulmonary fibrosis is a disease of aging, Hecker *et al.* [53] compared the capacity of young (two months) and aged (18 months) mice to repair bleomycin-induced lung injury and fibrosis. Although the severity of lung fibrosis in each group was similar at three weeks following bleomycin exposure, the aged mice were unable to resolve fibrotic lung injury at two months whereas young mice were largely free of fibrotic injury. Persistent lung fibrosis in the aged mice was characterized by the accumulation of senescent and apoptosis-resistant myofibroblasts, as well as sustained alterations in redox balance resulting from the elevated expression of NOX4 and an impaired capacity to induce the nuclear factor erythroid 2-related factor 2 (Nrf2)-mediated antioxidant response. Human IPF lung tissues also exhibited the imbalance between NOX4 and Nrf2, as well as NOX4 mediated senescence and apoptosis resistance in IPF fibroblasts. Genetic and pharmacological inhibition of NOX4 (with NOX4 siRNA and GKT137831) in older mice with established fibrosis attenuated the senescent and apoptosis-resistant myofibroblast phenotype and led to a reversal of persistent fibrosis. These findings implicating Nrf2 are in accord with prior studies showing that Nrf2 knockout mice are more sensitive to bleomycin and paraquat-induced lung injury than their wild-type counterparts, that primary lung fibroblasts isolated from IPF patients, as compared to healthy controls, have decreased Nrf2 expression and a myofibroblast (pro-fibrotic) differentiated phenotype, and that treatment with sulfaphane, an Nrf2 activator, increases antioxidant levels that results in decreased ROS levels, myofibroblastic de-differentiation, and TGF-β profibrotic effects. Although the role of the NOX4/Nrf2 pathway in AEC and mtDNA damage is unknown, these studies firmly suggest that the restoration of NOX4-Nrf2 redox imbalance in myofibroblasts may be an important therapeutic target.

Our group recently reported that mice globally deficient in *Ogg1^−/−^* are more prone to pulmonary fibrosis following asbestos exposure than their wild-type counterparts due in part to increased AEC mtDNA damage and apoptosis [98]. Interestingly, compared to AT2 cells isolated from WT mice, AT2 cells from *Ogg1^−/−^* mice have increased mtDNA damage, reduced ACO-2 expression, and increased p53 expression at baseline and these changes were augmented following crocidolite asbestos exposure for three weeks. Collectively, these data support a key role for AEC OGG1 and ACO-2 in the maintenance of mtDNA necessary for preventing AEC apoptosis and pulmonary fibrosis. These findings implicating AEC mtDNA damage signaling in mediating pulmonary fibrosis parallels work by other groups implicating mtDNA damage in the pathophysiology of diverse conditions, such as atherosclerosis, cardiac fibrosis/heart failure, diaphragmatic dysfunction from mechanical ventilation, and cancer [100,101,102,103,104,105,106]. Interestingly, mitochondria-targeted OGG1 diminishes ventilator-induced lung injury in mice by reducing the levels of mtDNA damage in the lungs [107]. Accumulating evidences also support an important association between p53, OGG1, and ACO-2, including (1) p53 regulates *OGG1* gene transcription in colon and renal epithelial cells [93]; (2) p53 deficient cells have reduced OGG1 protein expression and activity [93]; (3) p53 can reduce *Aco-2* gene expression [108]; (4) p53 activation is required for oxidant-induced apoptosis in *OGG1*-deficient human fibroblasts [93]; and (5) p53 sensitizes HepG2 cells to oxidative stress by reducing mtDNA [109]. Collectively, these data support a key role for p53 in modulating AEC mtDNA damage in the pro-fibrotic lung response following asbestos exposure that also has important implications for our understanding of the malignant potential of asbestos fibers. However, the precise molecular mechanisms by which OGG1, ACO-2, and p53 coordinately regulate mtDNA integrity in AEC as well as the translational significance of these findings in humans await further study.

A number of animal models of pulmonary fibrosis beyond the scope of this review have implicated excess plasminogen activator inhibitor (PAI-1) in augmenting AEC apoptosis. Shetty and colleagues have recently published a number of elegant studies showing an important dichotomous role of PAI-1 in promoting AEC apoptosis but reducing fibroblast proliferation and collagen production in the pathobiology of lung fibrosis [110,111,112]. Similar to older studies, these investigators showed that lung injury and pulmonary fibrosis are more evident in mice deficient in urokinase-type plasminogen activator (uPA), whereas mice deficient in PAI-1 are protected. To explore whether changes in AT2 cell uPA and PAI-1 contribute to epithelial-mesenchymal transition (EMT), AT2 cells from patients with IPF and COPD, and mice with bleomycin-, transforming growth factor β-, or passive cigarette smoke-induced lung injury all had reduced expression of E-cadherin and zona occludens-1, whereas collagen-I and α-smooth muscle actin (markers of EMT) were increased along with a parallel increase in PAI-1 and reduced uPA expression [110]. These studies suggest that induction of PAI-1 and inhibition of uPA during fibrotic lung injury promotes EMT in AT2 cells. These same investigators showed that fibroblasts isolated from human IPF lungs and from mice with bleomycin-induced lung fibrosis had an increased rate of proliferation compared with normal lung fibroblasts [111]. Basal expression of plasminogen activator inhibitor-1 (PAI-1) in human and murine fibroblasts was reduced, whereas collagen-I and α-smooth muscle actin were markedly elevated. In contrast, AT2 cells surrounding the fibrotic foci, as well as those isolated from IPF lungs, showed increased caspase-3 and PAI-1 activation with a parallel reduction in uPA expression. PAI-1 depletion and enforced expression studies in cultured fibroblast confirmed the inverse relationship between PAI-1 activation and collagen production. The authors suggested that depletion of PAI-1 in fibroblasts promotes an activated collagen producing cell that is resistant to senescence/apoptosis whereas activated PAI-1 augments AT2 cell apoptosis important for the propagation of lung fibrosis. Using a silica-induced model of lung fibrosis, these investigators showed p53-mediated changes in the uPA system promote lung fibrosis in part by reducing caveolin-1 scaffolding domain peptide (CSP), which is necessary for inhibiting p53 expression and silica-induced lung injury [112]. Notably, as compared to untreated WT mice, silica-exposed WT mice treated with CSP inhibited PAI-1, augmented uPA expression and prevented AEC apoptosis by suppressing p53. The authors suggested that silica-induced lung fibrosis is driven by important crosstalk between the p53-uPA fibrinolytic system in AT2 cells and provide support for a novel pharmacologic target (*i.e.*, CSP), in modulating this pathway. In contrast, another group working with murine fibroblasts recently showed that increased PAI-1 may drive age-related and bleomycin-induced pulmonary fibrosis at least in part by blocking fibroblast apoptosis [113]. Additional studies are required to better understand how precisely PAI-1 affects AEC and fibroblast mtDNA damage response, mitochondrial function and apoptosis as well as how this impacts lung fibrosis.

Mitochondrial ROS and mtDNA can also trigger NACHT, LRR, and PYD domains-containing protein 3 (NALP3) inflammasome signaling important in driving lung fibrosis following asbestos or silica exposure [37,57,114,115]. The mtDNA released into the circulation can act as a sentinel molecule triggering a DNA damage-associated molecular pattern (DAMP) that activates innate immune responses, especially toll like receptor (TLR)-9 signaling, leading to change a phenotype in lung fibroblasts and tissue injury, including lung fibrosis [12,116]. Gu and associates recently showed that intratracheal instillation of mtDNA into murine lungs triggers infiltration of inflammatory cells and production of inflammatory cytokines (*i.e.*, IL-1β, IL-6, and TNF-β) and that these effects were blocked when the lungs were pretreated with TLR-9 siRNA [117]. This suggests that the mtDNA DAMPs can activate innate immune signaling in the lungs via the TLR-9 pathway. Interestingly, Kuck and colleagues showed that mitochondria-targeted OGG1 protein infusion mitigates mtDNA DAMP formation and TLR-9-dependent vascular injury induced by intratracheally-instilled bacteria [118]. Using mice lacking vimentin, a type III intermediate filament, Dos Santos and colleagues demonstrated an important role for vimentin in regulating NLRP3 inflammasome signaling that promotes acute lung injury, IL-1β expression, alveolar epithelial barrier permeability, and lung fibrosis following exposure to lipopolysaccharide (LPS), crocidolite asbestos, and bleomycin [119]. Notably, a direct interaction between vimentin and NLRP3 was demonstrated as well as an important role for macrophages based upon the finding that bone marrow chimeric mice lacking vimentin have decreased lung fibrosis as well as levels of caspase-1 and IL-1β. Collectively, these recent studies provide insight into the role of mtDNA and vimentin in regulating the NLRP3 inflammasome signaling important in promoting lung inflammation and fibrosis.

## 4. The Role of Sirtuins in Mitochondrial Integrity, mtDNA Damage Repair, and Aging

### 4.1. Overview of the Sirtuin (SIRT) Family Members

The yeast silent information regulator protein (SIR2) is a highly-conserved protein that has been linked to increased longevity via maintenance of genomic stability in a variety of organisms, including *Drosophila melanogaster* and *Caenorhabditis elegans* [120,121,122]. The homologous mammalian sirtuin family (SIRTs) consists of seven identified members to date, which are localized to the nucleus (SIRT1, SIRT6, SIRT7), cytoplasm (SIRT2), and mitochondria (SIRT3-5), respectively. All sirtuins contain a conserved core domain with NAD^+^ binding activity, while most sirtuins catalyze NAD^+^-dependent deacetylation of lysine residues though SIRT4 is known to have ADP-ribosyltransferase activity and SIRT5 both desuccinylase and demalonyase activity. Members of the sirtuin family play a role in maintenance of genomic stability at multiple levels by participating in DNA repair, altering chromatin structure and function via histone deacetylation [120], and via adaptation of cellular metabolic flow and energy demand [121]. As such, sirtuins have been considered by many to be the guardians of the genome [122].

### 4.2. The Role of SIRTs and Mitochondria; Normal and General Diseases

Changes in mitochondrial number and function are implicated in the pathogenesis of aging and many of its associated diseases, such as Parkinson’s disease, presbycusis, diabetes and the metabolic syndrome, malignancy, and fibrosis. Though changes in mitochondria have been linked to many disease processes, the underlying detailed molecular mechanisms have yet to be fully elucidated as noted above. Major changes in the mitochondria occur with aging, including an increase in the Δψ_m_ with subsequent increases in ROS production and oxidative damage to mtDNA and other cellular macromolecules. Maintenance of intracellular redox balance is critical to cellular homeostasis and survival. Certain mtDNA haplogroups are associated with an increase in ROS production; these may be associated with a protective effect in early life due to protection against infection, though with age may in fact be maladaptive due to the presence of chronic oxidative stress [7].

Crosstalk between the mitochondria and nucleus has emerged as a potentially powerful regulator underlying age-related diseases. Using a mitochondrial cybrid model of age-related macular degeneration, Kenney *et al.* [30] showed varying bioenergetic profiles among mtDNA haplogroups and went further to demonstrate different gene expression profiles for both mitochondrial-encoded genes involved in cellular respiration and nuclear-encoded genes involved in inflammation and the alternative complement and apoptosis pathways. Based on their findings, they propose a constant interaction between the mitochondrial and nuclear DNA whereby the mtDNA haplotype sets a baseline bioenergetics profile for the cell which interacts with environmental factors to contribute to oxidative stress, mitochondrial dysfunction, cell death and disease. As extensively reviewed in detail elsewhere [18,123], considerable evidence demonstrate that preservation of the mitochondrial genome by various mechanisms beyond the scope of this review is critical for ensuring a functional mitochondria and, thereby, contributing to nuclear DNA stability and cell survival.

**Figure 3:**
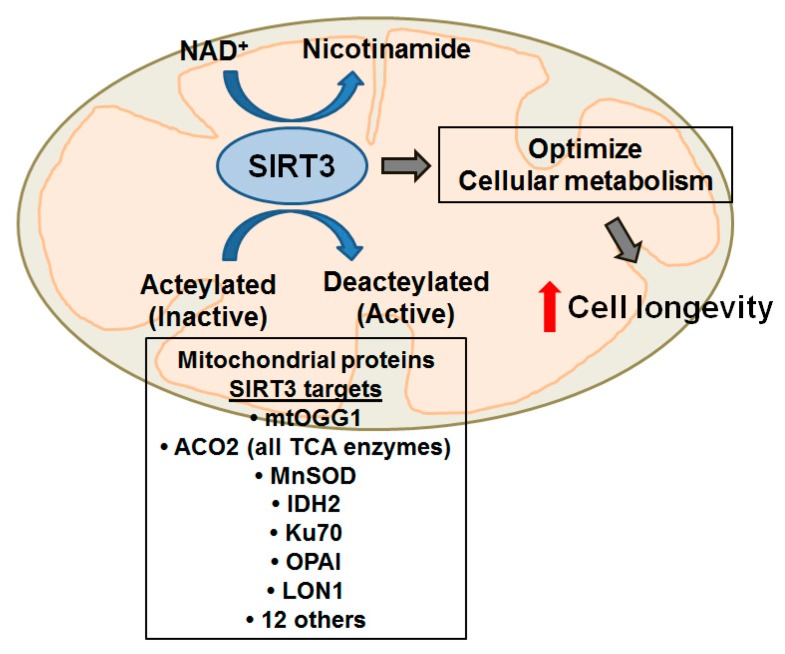
SIRT3 mitochondrial protein deactylation targets modulate cell metabolism and longevity. Red-up arrow, increase.

As emerging evidence reviewed above implicates the mitochondria as the sentinel organelle governing the cell’s cytotoxic response to oxidative stress in the lung, important questions emerge about how the redox balance in the mitochondria is maintained [12]. A crucial role of the mitochondria is to balance energy generation via oxidative phosphorylation with cellular nutrient supply; failure to do so may result in damaging levels or ROS, with resultant mtDNA damage and decreased mitochondrial biogenesis, or decreased cellular availability of ATP. SIRT3 has emerged as a key regulator in cellular ROS balance due to its role modulating mitochondrial homeostasis via deacetylation of multiple mediators of energy metabolism and cellular ROS, including members of the ETC, TCA cycle, and mitochondrial enzymes which detoxify ROS. As such, SIRT3 is considered the “gatekeeper” of mitochondrial integrity [124]. Figure 3 highlights some of the key mitochondrial SIRT3 proteins whose functions are post-translationally regulated by acetylation. Given the hypothesized relation between chronic oxidative stress, SIRTs, and aging [122,124], it is interesting that SIRT3 is the only sirtuin that has been closely associated with longevity in humans based upon the presence of a variable nucleotide tandem repeat (VNTR) enhancer within the SIRT3 gene that is associated with increased survival in the elderly [125].

### 4.3. The Role of SIRT3 in mtDNA Damage, Aging and Diseases Including Lung

As reviewed briefly above, repair of mtDNA damage following oxidative injury is a complex process that remains incompletely understood. Emerging evidence suggest an important role for SIRT3 in mtDNA repair. SIRT3 deaceylates and activates many mitochondrial proteins involved in energy metabolism (including all the enzymes involved in the TCA cycle such as ACO-2, isocitrate dehydrogenase 2 [IDH2], *etc.*), ETC members, antioxidant defenses, and mtDNA repair (OGG1) [121,124,126]. Murine SIRT3 depletion studies have shown that SIRT3 is necessary for cellular resistance against genotoxic and oxidative stress by preserving mitochondrial function and genomic stability. SIRT3 deficiency promotes intrinsic apoptosis by several mechanisms identified to date, including (1) augmenting mitochondrial ROS production due to acetylation and inactivation of manganese superoxide dismutase (MnSOD) and IDH2 [124,127]; (2) increasing cyclophilin D activity and down-stream mitochondrial permeability transition (MPT) [128,129]; (3) promoting Bax-mediated apoptosis by acetylating Ku70 [129]; and (4) attenuating p53-mediated growth arrest [130,131]. Notably, a recent study using glioma and renal epithelial tumor cells showed that mitochondrial OGG1 is a direct SIRT3 deacetylation target and that SIRT3 deficiency reduces the oxygen consumption rate (OCR) and 8OHdG mtDNA BER that increases mtDNA damage and intrinsic apoptosis [126]. Further, mtDNA mutations/deletions can reduce SIRT3 expression [132,133]. Although unclear in AT2 cells, loss of SIRT3 in keratinocytes augments ROS levels, reduces NAD^+^ levels, and promotes epidermal terminal differentiation [134]. These findings suggest SIRT3 may play an important role in maintaining AT2 cell mtDNA and cell survival in the setting of oxidative stress.

A decade ago, a novel function of ACO-2 was discovered, whereby this TCA cycle enzyme was also found to associate with mtDNA nucleoids and stabilize mtDNA in the setting of mtDNA instability [98]. ACO-2 activity is sensitive to the redox state of the cell, suggesting that in the setting of oxidative stress ACO-2 can be relocated from the TCA cycle to the nucleosome to aid in stabilization of the mtDNA with subsequent removal of oxidized Aco-2 by Lon protease [135]. As noted earlier, in an asbestos-induced model of pulmonary fibrosis, oxidant-induced mtDNA damage was blocked by overexpression of ACO-2, a mitochondria-targeted OGG1, and an OGG1 mutant incapable of DNA repair that chaperones ACO-2 [8,9]. The precise mechanism of mtDNA protection by mutant mt-OGG1 comparable to WT mt-OGG1 is not established but may involve blockade of oxidative modification sites on ACO-2, which are necessary for subsequent degradation by the Lon protease. Support for this possibility is that MG132, an inhibitor of Lon protease activity, prevents oxidant-induced reductions in ACO-2 activity in AECs [9]. Another possibility is via effects of SIRT3 on Lon protease since both proteins co-precipitate in breast cancer cells exposed to oxidative and hypoxic stress and SIRT3 silencing results in hyperacetylation and inactivation of Lon [136]. As activity of Lon can favor the transition from aerobic respiration to anaerobic glycolysis, regulate proteins of the TCA cycle and degrade proteins following oxidative damage, these findings suggest a unique coupling between Lon, SIRT3, OGG1, ACO-2, mtDNA repair and the metabolic state of the cell.

Chronic oxidative stress with resultant depletion of SIRT3 and perturbations in mitochondrial function and biogenesis has been increasingly identified as a vital element in the pathogenesis of age-related diseases. *Sirt3*-deficient *(Sirt3^−/−^*)mice have no obvious phenotype but are susceptible to age-linked diseases including metabolic syndrome, cancer, cardiac hypertrophy-fibrosis/CHF, hearing loss, acute renal injury, neurodegeneration, and radiation-induced fibrosis [124,137,138,139,140,141,142,143]. Notably, *Sirt3^−/−^* mice exposed to irradiation have reduced liver ACO-2 activity and increased mitochondrial p53 expression [140]. Aged mouse hematopoietic stem cells (mHSCs) have a diminished capacity to respond to oxidative injury induced by H_2_O_2_ resulting in decreased cell survival and increased apoptosis [144]. SIRT3 expression is reduced in aged mHSCs and SIRT3 overexpression in turn restored the proliferative ability of these cells. Whether SIRT3 affords similar changes in AT2 cells, the stem cell of the distal alveolar epithelium, is unknown. Interestingly, Huang *et al.* [145] showed that rat bone marrow mesenchymal stem cells can differentiate into AT2 cells and alleviate bleomycin-induced lung fibrosis when injected at the same time as bleomycin exposure. In skeletal muscle, aging is associated with oxidative damage to cellular macromolecules; exercise training has been associated with an increase in SIRT3 expression, in both young and old subjects, as well as reduced acetylation of IDH-2, a SIRT3 deacetylase target involved in cellular ROS detoxification [146]. In a murine model of acute kidney injury (AKI), cisplatin-induced oxidative injury decreased renal SIRT3 expression and increased mitochondrial fragmentation [141]. *Sirt3^−/−^* mice were more susceptible to cisplatin-induced AKI as compared to their wild-type counterparts and treatment with antioxidants restored SIRT3 expression and activity and protected against renal dysfunction in the wild-type mice but not in *Sirt3^−/−^* animals. In an *in vitro* cortical neuron model of oxidative stress, overexpression of SIRT3 increases mtDNA and mitochondrial biogenesis that has pro-survival effects [147]. In a rodent model of pre-diabetes, oxidative stress induced by a high-fat diet was associated with disruption of the SIRT3/PGC-1α axis with impairment in mitochondrial bioenergetics and decreased testicular mtDNA and adenylate energy charge [148]. A potential role of targeting SIRT3 in diabetes was supported by a recent study showing that overexpression of SIRT3 can mitigate palmitate-mediated pancreatic β cell dysfunction [149].

Murine models of SIRT3 deficiency confirm its protective role in multiple models of age-associated diseases related to oxidative stress. Lysine deacetylation has emerged as a key post-translational protein modification employed to activate mitochondrial signaling [150,151]. Mice deficient in SIRT3 exhibit striking increases in global protein acetylation; effects that were notably not seen in SIRT4- and SIRT5-deficient mice, suggesting that SIRT3 serves as the key mitochondrial deacetylase [152]. In a model of cardiac fibrosis, *Sirt3^−/−^* mice develop severe interstitial cardiac fibrosis and hypertrophy following application of a hypertrophic stimulus, whereas mice engineered to overexpress SIRT3 maintain normal cardiac structure and function [137]. These investigators showed that the mechanism of SIRT3 protection was by activation of the FOXO3a-dependent antioxidant genes catalase and MnSOD which reduced cellular ROS [137]. Another group recently showed that *Sirt3^−/−^* mice developed age-related cardiac dysfunction likely due to increased acetylation of various mitochondrial energy producing proteins resulting in myocardial energy depletion [153]. SIRT3 also seems important in pulmonary arterial hypertension (PAH) since pulmonary artery smooth muscle cells (PASMC) from patients with PAH display down-regulation of SIRT3; *Sirt3^−/−^* mice spontaneously develop PAH and SIRT3 restoration in a rodent model and *Sirt3^−/−^* mice PASMC attenuates the disease phenotype [154]. In a murine model of presbycusis, which is postulated to be due to chronic oxidative stress, decreased SIRT3 expression in the central auditory cortex was noted with concurrent increases in ROS, mtDNA damage, and SOD2 acetylation [155]. Taken together, these data strongly support the protective role of SIRT3 against oxidative stress in a variety of cell types and murine models of various degenerative diseases. Additional studies are necessary to better define the role of mtDNA integrity in mediating the beneficial effects of SIRT3 as well as the translational significance in humans, including pulmonary fibrosis.

### 4.4. Therapeutic Approach: Resveratrol/Viniferin/Honokiol

The first techniques used to enhance sirtuin expression involved caloric restriction due to the putative link between SIRT3 deacetylase activity and the metabolic state of the cell [124]. The role of SIRT3 in modulating mitochondrial energy metabolism will be discussed in detail the final section below. Exercise training has been associated with increased SIRT3 expression and recently has been linked to a decrease in mitochondrial protein acetylation, increased ACO-2 and MnSOD activity, and improved mitochondrial biogenesis in a cardiac model of doxorubicin-induced oxidative injury [156].

Recently, small molecule sirtuin inducers have been under investigation. Resveratrol (RSV), a naturally occurring polyphenol, extends the lifespan of diverse model organisms in a Sir2 (a silent information regulator)-dependent fashion [157]. RSV induces both SIRT1 and SIRT3 expression via stimulation of NADH dehydrogenases and mitochondrial complex I resulting in increases in the mitochondrial NAD^+^/NADH ratio [158]. Treatment with RSV has been shown to abrogate hyperoxia-mediated acute liver injury [158], airway remodeling and hyperreactivity in a model of allergic asthma [159], insulin resistance [160], and pulmonary fibrosis in a bleomycin mouse model [161]. Activation of SIRT3 via RSV in a murine model of cardiac fibrosis attenuated collagen deposition and cardiac hypertrophy via regulation of the TGF-β/Smad pathway; SIRT3 is required for the cardio-protective effects revealing a potential link between mitigation of oxidative stress and the signaling pathways responsible for fibrosis [142]. Viniferin, a natural dehydrodimer of RSV, may have increased antioxidant potential compared to RSV and has been shown to protect vascular endothelial cells from oxidative stress by suppressing intracellular ROS production [162]. Further, SIRT3 was shown to mediate the neuroprotective effect of viniferin in models of Huntington disease via an increase in the number of mtDNA copies, attenuation of the loss of mitochondrial membrane potential, and enhanced activity of MnSOD [163]. Pillai and colleagues showed that honokiol, a natural occurring compound from the bark of magnolia trees with anti-inflammatory, anti-oxidative, anti-tumor, and neuroprotective properties, reverses murine cardiac hypertrophy and fibrosis by a SIRT3-dependent mechanism [164]. Although it is unknown whether these small molecule sirtuin inducers are protective to AEC mtDNA and cell survival following exposure to oxidative stress, they represent an exciting therapeutic strategy for future studies addressing age-related diseases attributed to chronic oxidative stress, such as pulmonary fibrosis.

## 5. MtDNA and Metabolism in Mitochondria

### 5.1. Mitochondrial Metabolism—The Basics

As noted earlier, the mitochondria, long championed as the “powerhouse of the cell”, are now well-recognized as being critically important in overall cellular health, intracellular signaling, and disease pathobiology [12]. However, that does not diminish the importance of the organelle’s ATP-producing function, as mitochondrial metabolism is the main mechanism by which a cell synthesizes ATP. Mitochondrial metabolism is governed primarily by one aspect: the presence of oxygen. Under normal conditions, in the presence of oxygen, mitochondria utilize both oxygen and electrochemical gradients for the production of ATP via the ETC, known as oxidative phosphorylation (OXPHOS), and the TCA (or Krebs) cycle, while in the absence of oxygen the mitochondria rely on glycolysis and fatty acid oxidation for ATP production. Glycolysis produces pyruvate as an end product, which is then used by the TCA to produce the reducing equivalents (e.g., NADH and FADH_2_) necessary for the ETC to convert ADP to ATP. Fatty acid oxidation, which occurs in the mitochondrial matrix, breaks fatty acids down from triacytlglycerols by β-oxidation and forms both acetyl-CoA (which is converted to citrate in the TCA cycle) and ATP [165]. The interaction between cytosolic liquid triacytlglycerol droplets and mitochondria in fatty acid oxidation must maintain a fine balance, as lipids can either inhibit or uncouple OXPHOS [166]. These anaerobic cycles produce less ATP per cycle, but can be much faster making them indispensable for cells that are not always under “ideal” conditions (*i.e.*, oxidative stress). Cells typically utilize these anaerobic pathways under low-oxygen conditions or when the cell requires energy faster than can be produced by O_2_-dependent means. However, mitochondrial damage, be it due to mtDNA damage, ROS, or ETC complex inhibition, can lead the cell to favor these alternate ATP producing pathways. The Warburg effect, or aerobic glycolysis, is where the cell favors the glycolytic pathway over the ETC even in the presence of O_2_, a phenomenon nearly synonymous with a cancerous phenotype as it can rapidly produce copious amounts of ATP, which are useful for the rapid growth of cancer cells [167]. However, excessive glycolysis, beyond that which is utilized by cancerous cells, can promote cell stasis, excessive mtDNA damage, and cytotoxicity [168].

### 5.2. Role of Mitochondria-Derived ROS, mtDNA Damage, and Mitochondrial Metabolism

ROS, especially O_2_^•−^, is a natural byproduct of the ETC due to inefficiencies in electron transfer between the four complexes (I, II, III, and IV). ROS are not inherently detrimental, but increased mitochondrial metabolism or damage within the OXPHOS pathway (*i.e.*, buildup of acetylated metabolic intermediates, ETC complex damage) increases cellular ROS, and, as detailed above, can cause mtDNA damage and harm components of the ETC, which further promotes ROS production. NAD^+^, a natural byproduct of metabolism, is produced when NADH is reduced by electrons from the ETC; with an impaired ETC, cells instead use glycolytic pathways or fatty acid oxidation and, as such, metabolic reducing equivalents (*i.e.*, NADH) build up in the mitochondria, reducing NAD^+^ formation and down-stream NAD^+^-dependent processes (e.g., SIRT3 deacetylation—see below) [165]. Decreased NAD^+^ levels promote OXPHOS dysfunction leading to metabolic failure in cells, which is a common phenotype in the aged [169]. However, strategies to augment NAD^+^ levels can prevent apoptosis-induced complex I inhibition, the depletion of intracellular ATP, and preserve mtDNA integrity, possibly by preventing an increase in BAX expression (6). Additionally, ACO-2, a TCA cycle intermediary metabolite, can stabilize the mtDNA and, thereby, linking mtDNA integrity with metabolic efficiency [135]. However, the mechanism by which ACO-2 toggles between these two ACO-2 functions is not well understood. Notably, mutations in *ACO-2* gene can cause syndromic optic neuropathy with encephalopathy and cerebellar atrophy in humans [170].

Disruption of mitochondrial dynamics is an early event in ROS-induced intrinsic apoptotic cell death. Excessive ROS production can induce ER Ca^2+^ release, reduce the cell’s abilty to produce autophagosomes, and contribute to degenerative diseases via effects on mtDNA [12,171]. OXPHOS dysregulation arising from mtDNA mutations/damage results in an increase in the invasive cell phenotype via effects on matrix metalloproteinase (MMP) family members. It also mimics lack-of-oxygen by causing increased lactate production and acidification of the extracellular environment, and can act parallel to (and independent of) hypoxia inducible factor (HIF)-1α activation to increase angiogenesis and glucose uptake [172]. Inflammatory signaling typically increases oxygen consumption (OCR), ROS production, and Δψ_m_, but does not significantly affect spare respiratory capacity (SCR—the difference between a cell’s maximal, uncoupled respiration, and its normal, basal respiration) when normalized to mitochondria content [173]. With progressive cellular stress, mitochondrial rupture and release of mtDNA can occur, which can lead to TLR-9/NLRP3 inflammasome activation and a vicious inflammatory cycle [115,117].

Mitophagy is the cellular mechanism in place to discard damaged and ineffective mitochondria with extensive mtDNA damage [3,174]. Healthy mitochondria can fuse, and such fusion acts as a buffer against mitophagy, ensuring that, optimally, fully-functional mitochondria will be spared, while those mitochondria that cannot fuse will be more easily mitophagized [3,174]. Full mitochondrial fusion can also allow for mtDNA and nucleoid transfer, thereby diminishing/alleviating mtDNA damage. However, fusion can still occur in mitochondria with extensive mtDNA damage if initiated by a healthy mitochondrion, which can revive the metabolic functioning of the damaged mitochondrion. Metabolically inefficient mitochondria that overexpress mitofusion genes can escape mitophagy [174]. Some inherited mitochondrial haplotypes display significant differences in ATP and ROS production and ETC complex expression; those that exhibit relatively lower productions of ATP and ROS appear to be more inclined toward dysfunction and resultant disease independent of nuclear DNA mutations [30]. As reviewed in detail elsewhere [7], there has been extensive study of mitochondrial lineage and how the inheritance of mitochondrial mutations can affect disease progression and severity. Notably, altered proteins resulting from damaged nDNA can allow mtDNA damage to accumulate [7,12]. For example, PINK1 and PARK2 mutations prevent the proper functioning of autophagosomes thereby preventing damaged mitochondria from being destroyed, leading to even greater mtDNA damage.

Although the role of mitochondrial metabolic pathways in regulating AEC function and maintaining mtDNA integrity are not well established, there is some information in other cell types. For example, in Huntington’s disease there are increased numbers of large, unhealthy mitochondria with decreased Δψ_m_, PGC-1α, ATP production, ETC function, ADP-uptake, glucose metabolism, and SRC [175]. PGC-1α, a regulator of both metabolism and mitochondrial biogenesis, is upregulated by RIP1 (receptor-interacting protein 1); a decrease in RIP1 leads to an increase of dsDNA breakage and oxidative glycolysis, a reduction in the NAD^+^ pools, and suppression of cell proliferation [168]. RIP1 maintains cancer cell glycolytic metabolism such that enhanced cell proliferation can occur, but its loss results in excessive glycolysis and p53-mediated cell stasis. Hyperglycemic damage in diabetic heart cells can cause alterations to mtDNA as a result of alterations in the supply of metabolic substrates (*i.e.*, NAD(P)^+^/NAD(P)H, GSH/GSSG, and TrxSH_2_/TrxSS) that can affect mitochondrial health [166]. Using a diabetic heart mouse model, these same investigators showed that cardiac muscle contraction and Ca^2+^ signaling are reduced largely because of alterations in complex I resulting from mitochondrial metabolic damage. Impaired pulmonary artery endothelial cell regeneration is linked to mitochondrial DNA deletion, and hypoxia-reoxygenation reduces p53, PGC-1α, ATP, Δψ_m_, caspase-induced apoptosis, the levels of mitofusin 1 and 2, and mitophagy [176]. Hypoxic conditions also render significant damage to mtDNA, through increased complex III O_2_^•−^ production [12]. Lung fibrosis in bleomycin-treated rats is associated with significant mtDNA damage, dysfunction of mtDNA-encoded ETC subunits, and increased ROS production and TGFβ levels [36]. Although the primary cell type mediating these effects was not identified, future studies focusing on mitochondrial metabolism in AECs will be of considerable interest. We reason that AEC mtDNA damage that accumulates over time can promote “aged” AT2 cells that are more prone to triggering pulmonary fibrosis following an environmental or oxidative stress insult.

### 5.3. Role of Sirtuins and Energy Metabolism in Mitochondria

Convincing evidences implicate SIRT-induced mitochondrial dysfunction in aging as well as diseases associated with aging (*i.e.*, cancer, neurodegeneration, CHF, and metabolic syndrome/diabetes) and will likely prove important in the pathophysiology of lung disease, such as IPF, asbestosis, and lung cancer [177,178,179]. The paradigm that is emerging by which SIRT3 affects cell metabolism and longevity is illustrated in Figure 3. As noted above (see Section 4), SIRT3 deaceylates and activates numerous mitochondrial proteins involved in diverse functions involving mitochondrial energy metabolism (including all the enzymes involved in the TCA cycle such as ACO-2, *etc.*), ETC members, antioxidant defenses, and mtDNA repair. Global loss of the *Sirt3* gene augments mitochondrial ROS levels, reduces OCR, NAD^+^ levels, and ATP production, and promotes radiation-induced genotoxic stress response and a tumor permissive phenotype [124,126,127,180,181]. There is a negative correlation between ROS generated by complex 1 in the ETC and lifespan, and bypassing complex 1 increases lifespan in the fruit fly by mechanisms that are uncertain, but may involve altered sirtuins and cellular NAD^+^ levels [178,182]. Given the “mitochondrial gatekeeper” role of SIRT3 for post-translational modification of mitochondrial proteins, not surprisingly SIRT3 is implicated in modulating mitochondrial metabolism. For example, Bass and colleagues demonstrated that circadian control of SIRT3 activity resulted in rhythms in the acetylation and activity of oxidative enzymes and respiration in isolated mitochondria, and that NAD^+^ supplementation restored mitochondrial protein deacetylation and augmented OCR in circadian mutant mice [183]. Mitochondrial NAD^+^ levels are also regulated by SIRT3 activity [182]. Lower levels of NAD^+^ due to aging have also been found to produce a hypoxic-like state that inhibits intracellular signaling [184]. There is some evidence that simply replacing the cellular NAD^+^ pool promotes recovery of cells from the aging metabolic phenotype [169]. PGC-1α-dependent SIRT3 signaling prevents complex 1 damage and preserves ETC function [185]. Increased PGC-1α levels have also been implicated in mitochondrial biogenesis, via a serine protease Omi-dependent mechanism [186]. Loss of SIRT3 can exacerbate the Parkinson’s phenotype in mice through inhibition of complex 1, leading to excess ROS production and mtDNA damage, resulting in neuronal death [143].

Other SIRTs, especially SIRT1, may also have important roles in mitochondrial metabolism. Cells and tissues from old animals exhibit an increase of SIRT1 expression that is associated with decreased SIRT1 activity, NAD^+^ levels, and ETC activity and augmented levels of nuclear p53, PARP, FOXO1, and oxidation of proteins, DNA, and lipids [178,187,188]. Despite SIRT3’s key mitochondrial function, there is some evidence suggesting that its activity is highly dependent upon the activity of the nuclear-localized SIRT1 effects on RELB [173]. Additional studies are necessary to better understand the roles of SIRT3 and other sirtuins on AEC mitochondrial metabolism in the setting of oxidative stress as well as the translational significance in human lung diseases.

## 6. Conclusions

The available evidence reviewed herein convincingly shows that AEC mtDNA plays a key role in modulating mitochondrial function and apoptosis. Furthermore, AEC mtDNA damage following oxidative or environmental stress may be important in promoting pro-fibrotic signaling as can be seen in IPF, asbestosis, and other fibrotic lung diseases. A hypothetical model of AEC mtDNA damage in mediating intrinsic apoptosis that we reviewed is shown in Figure 2. Furthermore, we explored the data implicating that ongoing mtDNA damage may also be important in promoting other degenerative conditions, aging, and cancer (Figure 1). Emerging findings also suggest that SIRT3 and mitochondrial metabolism may be important in attenuating the deleterious effects of oxidative stress in a variety of cell types and murine models of degenerative diseases. Thus, the mitochondria clearly function not only as the “powerhouse” of the cell, but also regulate important cellular functions and signaling that determine cell life and death decisions. The role of OGG1, ACO-2, and small molecule SIRT3 inducers in modulating mtDNA damage in AEC as well as other cells and how this translates into disease pathophysiology await further study. We reason that the OGG1/ACO-2/SIRT3/mtDNA axis is important in regulating complex cell signaling that promotes pulmonary fibrosis as well as other degenerative disease of the lungs and tumor development.
